# Innovative QR Code System for Tamper-Proof Generation and Fraud-Resistant Verification

**DOI:** 10.3390/s25133855

**Published:** 2025-06-20

**Authors:** Suliman A. Alsuhibany

**Affiliations:** Department of Computer Science, College of Computer, Qassim University, Buridah 51452, Saudi Arabia; salsuhibany@qu.edu.sa

**Keywords:** barcode fraud, secure barcode, watermarking, object detection, information security, neural network

## Abstract

Barcode technology is widely used as an automated identification system that enables rapid and efficient data capture, particularly in retail environments. Despite its practicality, barcode-based systems are increasingly vulnerable to security threats—most notably, barcode substitution fraud. To address these challenges, this paper presents an innovative system for the secure generation and verification of Quick Response (QR) codes using a digital watermarking technique. The proposed method embeds tamper-resistant information within QR codes, enhancing their integrity and making unauthorized modification more difficult. Additionally, a neural network-based authentication model was developed to verify the legitimacy of scanned QR codes. The system was evaluated through experimental testing on a dataset of 5000 QR samples. The results demonstrated high accuracy in distinguishing between genuine and fraudulent QR codes, confirming the system’s effectiveness in supporting fraud prevention in real-world applications.

## 1. Introduction

The evolution of market selling systems has been significantly influenced by advancements in real-time data entry technologies, which have replaced manual processes that were prone to inefficiency and human error. Among the most transformative tools in this space is barcode technology—a widely adopted method for automatic identification and real-time data capture. These systems enable rapid item recognition at checkout, improving operational speed and reducing customer wait times.

Barcodes, particularly Quick Response (QR) codes, are encoded optical patterns that store digital information. Initially developed by Denso Wave in 1994 for automotive tracking, QR codes have since become ubiquitous, used across industries such as retail, healthcare, logistics, and marketing due to their ability to hold extensive data in a compact form and their compatibility with smartphone scanners. Unlike one-dimensional barcodes, QR codes encode data both horizontally and vertically, offering greater information density and faster readability.

However, the widespread deployment of barcode systems has raised critical security concerns. Barcode substitution fraud—where genuine barcodes are replaced with forged ones to manipulate product information or pricing—poses a growing threat. This type of fraud not only results in financial losses but also erodes consumer trust and demands increased manual oversight. Studies have identified vulnerabilities in conventional barcode systems stemming from the ease of barcode regeneration, insufficient authentication protocols, and the absence of robust tamper-proof features.

Recent approaches to mitigate barcode-related fraud have included the integration of sensor-based object detection, visual signature matching, and machine learning-based image verification systems. Despite these advances, most solutions require complex infrastructure, significant computational resources, or cloud-based authentication platforms—challenges that limit their scalability and adoption in cost-sensitive environments. Moreover, these methods often lack localized, market-specific protection mechanisms.

To address these gaps, this paper introduces a novel system for tamper-proof QR code generation and fraud-resistant verification. The core of the proposed method lies in embedding secure digital watermarks within the QR codes using a lightweight technique that preserves their readability. Complementing this, a neural network-based authentication model is employed to verify the legitimacy of scanned codes in real time, without requiring external storage or cloud-based matching.

The proposed system aims to provide a scalable and practical solution to QR code authentication, suitable for deployment in environments with limited computational resources. Its design emphasizes local validation, market-specific watermarking, and resistance to reprinting or cloning attempts. The system’s performance is validated through extensive experimentation on a dataset comprising 5000 QR samples.

The remainder of this paper is organized as follows: Section 2 reviews related work and existing techniques for barcode fraud prevention; Section 3 demonstrates the problem through real-world examples; Section 4 describes the proposed secure QR generation and verification system; Section 5 details the implementation and experimental setup; Section 6 discusses the results and future directions; and Section 7 concludes the paper.

## 2. Related Works

This section explores prior research that has proposed various solutions to address barcode substitution fraud and provides an overview of existing barcode authentication systems.

### 2.1. Barcodes: An Overview

Barcodes are traditionally defined as machine-readable representations of data in the form of visual patterns that can be decoded using optical devices. However, alternative definitions have emerged in the recent literature, particularly in the context of sensor integration. For example, barcodes are increasingly viewed as passive data carriers that interact with sensing technologies such as optical scanners, RFID systems, and camera-based readers to enable automatic identification and tracking. This perspective emphasizes the role of barcodes within sensor-based systems, where they function not only as data encoders but also as triggers or elements in broader data acquisition and monitoring frameworks [1,2].

There are three primary types of barcodes: one-dimensional, two-dimensional (2D), and three-dimensional barcodes. According to [3], “one-dimensional barcodes are arrays of parallel lines that carry pattern information.” The 2D barcode was developed to store more information. Represented as an optical marker, it can store significant amounts of data in a small area without requiring access to an external database; however, more advanced readers, such as mobile phones, are needed. As noted in [4], 2D barcodes are a cost-effective tool for advertising and marketing, providing access to detailed information about a product, guiding navigation within buildings, and facilitating mobile phone payments and device authentication. The three-dimensional barcode, while similar to the 2D barcode, incorporates colors [5]. It is also embossed on the product, with the scanner analyzing its height to decode the data [6].

In recent advancements, the integration of barcodes with sensor technologies has become increasingly prominent, particularly within smart environments and industrial applications. Barcodes serve as data-rich identifiers that can be detected and interpreted by sensor-based systems, such as optical scanners, camera modules, or embedded vision sensors. These sensors act as data acquisition interfaces that capture the barcode image, process it in real time, and transmit the decoded information for subsequent actions such as inventory tracking, authentication, or environmental monitoring. This sensor-barcode interplay enables automated decision-making and efficient data flow in domains like supply chain management, healthcare, and intelligent retail systems. Therefore, barcodes function not merely as static labels but as dynamic components within sensor-driven infrastructures.

### 2.2. Barcode Applications Across Industries

Barcodes are widely adopted across a variety of industries due to their efficiency, low cost, and ease of implementation. While they are extensively used in retail for inventory management and point-of-sale operations, their applications extend far beyond. In transportation and logistics, barcodes play a critical role in package tracking, shipment verification, and supply chain management. Similarly, in wholesale operations, barcodes are used to streamline bulk transactions and inventory control. In these sectors, the value associated with each transaction is typically higher, making barcode systems more vulnerable to fraudulent activities, such as duplication, tampering, and unauthorized product substitution. As such, securing barcode systems in high-stakes environments is imperative to ensure trust, operational continuity, and customer safety.

### 2.3. From Traditional Barcodes to QR Codes

Barcodes have long served as a foundational technology for encoding and retrieving data across numerous applications. As the demand for greater data capacity, faster scanning, and enhanced versatility grew, QR (Quick Response) codes emerged as a two-dimensional evolution of traditional linear barcodes. Unlike 1D barcodes, which store limited information along a single axis, QR codes encode data in both vertical and horizontal dimensions, allowing them to store significantly more information—such as URLs, authentication keys, or encrypted payloads. This advancement enables QR codes to support a broader range of applications, from mobile payments and secure authentication to smart logistics and traceability systems. The transition from barcodes to QR codes represents a natural progression in the pursuit of secure and efficient data transmission, particularly in sensor-integrated environments where dynamic and high-density data encoding is essential.

Previously, a set of approaches to mitigate barcode-related fraud have included the integration of sensor-based object detection [7] and visual signature matching [8]. Moreover, several studies on methods for preventing barcode fraud were reviewed in [9]. The traditional verification approach involves the cashier at the retail checkout reading the product description displayed on the screen and visually matching it with the actual product. This method is less effective, time-consuming, and dependent on human accuracy.

Other techniques focus on the non-price characteristics of products, such as those used in self-checkout systems, where the weight of a scanned item is verified against the actual weight of the item placed in the bag. However, in many cases, the system fails to detect fraud when two products share the same weight. To address this, another method checks both the weight and shape of the product. While this provides an extra layer of security, it remains impractical when two products have identical characteristics.

The third characteristic that can be used for verification is color, where the product’s color is compared with the color associated with the scanned barcode. However, this method may be less effective if both the product and barcode are the same color. It can also be influenced by factors such as lighting, viewing angle, and camera distance. An alternative approach involves using a visual signature, where an image of the product and barcode is captured. The system then compares the visual signature of the product with a stored model linked to the barcode. This visual signature can encompass the color, shape, and texture.

The features of image processing, such as background subtraction, color analysis, motion correction, and image clean-up, have been shown to vary in effectiveness [9,10,11,12,13]. A study by [14] explored the issue of fraud prevention, which typically involves the system generating geometric features from a product image and retrieving the corresponding feature data associated with the scanned barcode from a feature database. These generated geometric features are then matched with the retrieved ones based on geometric transformation. If the number of matching features falls below a predetermined threshold, the system triggers an alert. However, several drawbacks of these methods were highlighted in [15]. For example, because this approach relies on feature object identification from product images, it demands significant resources, large databases, and incurs high hardware costs [16]. Additionally, it introduces a specular reflection problem, which can interfere with the identification system. The method also requires several image processing steps, including ensuring that images have identical resolutions, removing pixels from the current image after performing pixel-by-pixel comparisons with the reference image, and applying image feature identification algorithms to detect the presence or absence of features in one or more product images.

### 2.4. QR: An Overview

QR codes are graphical representations of data that store information in a two-dimensional matrix. They have become more and more ingrained in our daily lives over the past several years. Their widespread use in sectors ranging from marketing to logistics underscores both their versatility and utility. According to the QR Code Trends Report by Uniqode [17], QR code scans increased by 57% globally from 2021 to 2022 across 50 countries. Notably, the report documents 32 million QR code scans during this period. While this figure highlights a significant level of adoption, more recent industry estimates suggest a substantially higher volume of active barcodes in circulation. For instance, GS1, the global standards organization for barcoding, reports that billions of barcode scans occur daily across retail, logistics, and healthcare sectors. This widespread usage underscores the critical importance of securing barcode systems against fraudulent reproduction and unauthorized manipulation, particularly in high-volume environments such as retail chains, transportation, and wholesale operations. This exponential growth can be attributed to several factors, including the global COVID-19 pandemic, which accelerated the adoption of contactless technologies. Additionally, the rise in mobile usage and advancements in QR code technology have contributed to their widespread acceptance. QR codes continue to evolve as a powerful tool for bridging the physical and digital worlds, with the industry witnessing a remarkable 323% rise in QR code generation from 2021 to 2023, as indicated by their survey [17].

QR codes have become suitable for a wide range of applications, serving a multitude of purposes with their ease of utilization and cost-effectiveness. As a result, they have notably become the preferred choice for advertisers looking to connect with potential customers across various platforms. Facilitating the rapid dissemination of information through Uniform Resource Locator (URL) encoding stands as one of the most prevalent use cases. Despite their convenience, QR codes have been exploited by cybercriminals who embed fraudulent links, which can be difficult to distinguish from legitimate ones, leading to malware installation or phishing redirection. This misuse underscores the need for attentiveness, as users may unintentionally scan malicious QR codes, posing a risk to the security of scanning devices, leading to substantial financial losses and significant data breaches.

Several studies have investigated the security vulnerabilities and forgery detection mechanisms associated with barcode and QR code technologies. For instance, ref. [17] examined the use of watermarking in printed codes to deter counterfeiting, while [18] explored the integration of cryptographic features into QR codes for enhanced authentication. Additionally, [19] presented a machine learning-based approach to identify forged QR code patterns under various distortion conditions. Recent works, such as [20,21], further highlight the applicability of deep learning models in real-time barcode verification and tampering detection. These studies collectively underscore the increasing reliance on intelligent, image-based techniques to address the limitations of traditional barcode security mechanisms.

A very recent study in [22] proposed QR Shield, which is a dual machine learning-based system designed to identify and detect malicious links embedded within QR codes. By utilizing a benchmark dataset of URLs, QR Shield effectively analyzes and classifies the embedded links as either safe or malicious. The system’s performance was evaluated using four metrics, and the experimental results demonstrated a high accuracy rate of 96.8%, showcasing its potential for enhancing the security of QR code scanning processes.

### 2.5. Insights from Related Studies

The escalation of URL-based attacks prompts an examination of the motivations behind these criminal activities and the significant human factors and psychological aspects that contribute to their success [23]. Krombholz et al. [24] highlight the susceptibility of QR codes to various forms of cyberattacks, such as phishing, due to their role in facilitating content accessibility. Ji’s study [25] explores their diverse applications, ranging from advertising to mobile payments, and also highlights the potential for forgery. Furthermore, Wahsheh and Luccio [2] conduct an in-depth analysis of the security services surrounding QR code scanners by analyzing 100 code scanner applications and evaluating their security features. They categorize these applications based on the level of security they provide and offer recommendations for developers to create code scanner applications that are both usable and secure. Collectively, these studies underline the crucial need to balance the usability of QR codes with effective security measures to ensure safe digital communication.

Moreover, a study in [26] proposed a novel approach to protect QR codes from tampering, consisting of two main stages: embedding a security feature within the QR code and evaluating the similarity between the QR code on the product and authentic ones. To address the first challenge, error-correcting codes are employed to encode and decode the embedded feature, ensuring robustness against errors in noisy communication environments. A deep neural network is utilized to integrate and extract the encoded data from the QR code, demonstrating resilience to real-world distortions caused by printing and photographing processes. For the second challenge, a Siamese network architecture is applied to assess the similarity between QR codes effectively.

A study in [27] utilized blockchain-based systems to create a decentralized and secure platform, ensuring product authenticity and reducing counterfeiting risks. By integrating blockchain technology with QR codes, manufacturers can safeguard their brands, enhance consumer trust, and uphold a secure and transparent supply chain. Also, in [28], a framework leveraging blockchain technology to enhance the detection of counterfeit products is proposed. The framework provides a comprehensive and secure approach to tracking the journey of items throughout the supply chain.

An advanced approach for QR code extraction, termed Adaptive Morphological Contour-Based QR Code Extraction (AMCQE), is proposed in [29]. The method follows a structured five-step process: grayscale conversion, Gaussian blurring, thresholding, contour detection, and morphological closing. These steps effectively enhance QR code detection in images with complex backgrounds and noise. Furthermore, a robust QR code verification system is introduced using Mobile-ViT, a lightweight transformer-based architecture that leverages global representation, and learning to accurately differentiate between legitimate and counterfeit QR codes. Experimental results highlight the method’s effectiveness, achieving a high accuracy of 99.28% with a processing time of just 0.08 s, demonstrating its practicality for real-world applications. Furthermore, a study in [30] introduces an innovative approach for reliable product authentication using QR code technology enhanced by mathematical computational algorithms. Through the implementation of this system, customers can identify counterfeit goods and help preserve brand integrity. The proposed method involves generating unique QR codes for each product, embedding encrypted information such as product numbers, manufacturing dates, and batch numbers. These details are hashed and encrypted using advanced computational algorithms to ensure security. The system employs two algorithms: one for generating the QR codes with product-specific information and another for verifying the authenticity of the product via a mobile application, ensuring consumers can confirm that they possess a genuine item.

QR code scanning relies heavily on various types of sensors, including optical sensors in smartphones, barcode readers in retail systems, and industrial scanners used in logistics and supply chains. These sensors capture visual data from QR codes under diverse lighting, angle, and motion conditions. Our proposed AI-based watermarking approach directly enhances the reliability of sensor-mediated QR code authentication by embedding imperceptible security features that remain detectable across different sensor capture environments. By doing so, it mitigates the risk of sensor manipulation, spoofing, or misreads caused by environmental interference or high-quality forgeries. As QR scanning sensors are increasingly integrated into IoT systems and smart infrastructures, securing these sensor operations becomes critical. Our system contributes to this goal by ensuring that even if the scanning is performed via basic optical sensors, authentication remains resilient due to embedded watermark verification and machine learning analysis.

### 2.6. Our Contribution

Recent advancements have explored watermarking as a means of securing QR codes by embedding imperceptible or visible patterns to aid in authentication and tamper detection. Techniques such as frequency-domain watermarking, spatial blending, and color-based encoding have been proposed to hide information within the QR code without compromising its scannability. These methods provide a layer of verification, especially when QR codes are printed and susceptible to physical duplication or manipulation.

However, many of these approaches require specialized hardware, external verification infrastructure, or are vulnerable to high-quality forgeries that replicate both the QR content and the watermark. In contrast, our proposed system introduces a visually embedded watermark pattern that is perceptible to the human eye but challenging for automated systems to replicate accurately. By combining this approach with a convolutional neural network (CNN)-based classifier trained to recognize subtle discrepancies introduced during forgery, our method achieves secure and efficient local authentication without reliance on external databases or cloud verification. This integration enhances the robustness of printed QR codes against fraudulent reproduction.

Our contribution is the introduction of a secure QR that cannot be regenerated, utilizing a digital watermarking technique while preserving the essential features of the QR, demonstrating its cost-effectiveness. To read this secure QR, we developed a novel authentication system based on a neural network. The system was subsequently evaluated through experimental testing. One of the key contributions of our work is the integration of artificial intelligence through a convolutional neural network (CNN) model to classify and authenticate QR codes based on visually embedded watermark patterns. Unlike traditional systems that rely on visual inspection or static matching, our approach leverages deep learning to dynamically detect forged QR patterns with high accuracy, enhancing both the robustness and adaptability of the authentication process.

## 3. Problem Demonstration

The barcodes currently in use in markets are susceptible to various straightforward and easily executed attacks. To illustrate this, several one-dimensional barcodes commonly used in markets were experimentally analyzed for two major fraud issues: duplication and swapping. Various tools were employed for this experiment, which will be detailed in the following sections.

### 3.1. Experiment of Duplication Fraud

The duplication fraud issue refers to the ability to create a copy of an existing barcode that can be read by the system. A range of commercial and free tools can be used for this purpose, including iBarcoder [31], Easy Barcode Creator [32], and TEC-IT tools [33]. These tools offer a variety of settings and options for barcode generation, allowing users to not only create barcode objects but also add text, image boxes, and graphics. Using these tools, we regenerated different barcodes collected from various markets, as listed in Table 1. Once regenerated, these barcodes were processed through the system, and the results confirmed that they were successfully readable.

### 3.2. Swapping Fraud Experiment

Swapping fraud involves copying existing barcodes and replacing them with others, enabling the manipulation of product prices by swapping barcodes between items. This practice results in duplication fraud, as barcodes are exchanged to alter the pricing of products associated with the original barcode.

In our experiment, we duplicated two different barcodes for two selected products and swapped their labels by printing and affixing the regenerated barcode to each product. At checkout, the system read the swapped barcodes as if they belonged to the original products. While this type of fraud has been studied before, as seen in [34], it motivated us to explore a secure barcode solution that could help prevent such fraudulent activities.

## 4. Materials and Methods

This section details the architecture, components, and experimental procedures used to develop and evaluate the proposed fraud-resistant QR code generation and verification system. In particular, the methodology comprises five phases: (1) the architecture of the proposed system, (2) QR code dataset generation, (3) the workspace configuration, (4) data training and testing, (5) the classifier–reader integration, and (6) evaluation and analysis.

### 4.1. The Proposed QR System

The proposed QR system recognizes that most QRs currently used in markets can be easily regenerated using commonly available tools. Furthermore, many of these QRs are not market-specific, enabling different markets to use the same QR generator. To address these issues, the proposed QR system was designed to generate QR codes that are resistant to regeneration and unique to each market. The details of this proposed QR system are explained in the following sections.

#### 4.1.1. Secure QR Code Generation with Watermarking

To strengthen resistance against QR code cloning and tampering, we implemented a lightweight spatial-domain digital watermarking technique integrated directly into the QR code generation process. Unlike one-dimensional barcodes, QR codes offer a greater capacity for information encoding and do not require backend database access, making them ideal for standalone authentication in constrained environments [4].

**Figure 1:**
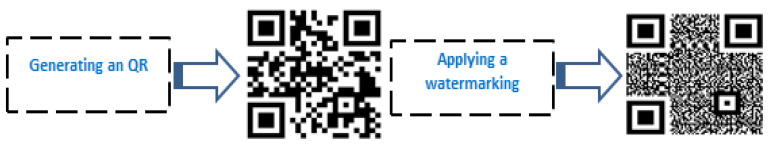
QR code with watermark generation phase.

The generation process, as shown in Figure 1, proceeds as follows:

First, a standard QR code is created using an encoder that includes alignment patterns to enhance the scanning stability and data recovery. Next, a unique anti-counterfeiting watermark pattern—distinct for each market—is embedded into the QR image using a least significant bit (LSB) modification technique. This embeds watermark data by altering the least significant bits of pixel values in the image. This method is commonly used due to its simplicity and minimal impact on image quality [35]. This also allows the secure insertion of imperceptible patterns within the spatial domain of the QR image without visibly altering its structure or compromising its readability.

The embedded watermark serves multiple purposes: it provides a unique identity per issuing market, enables tamper detection during scanning, and verifies the authenticity of the code against fraudulent reproduction. The technique was specifically chosen for its robustness against common distortions such as printing, resizing, and scanning—making it practical for real-world applications.

It is important to emphasize that each market must apply a different watermark pattern when generating its own QR codes to preserve the uniqueness and traceability required for secure authentication. Moreover, the embedded watermark is not extracted explicitly during the verification phase. Instead, the entire QR code image—including the embedded watermark pattern—is processed directly by the classification model. The watermark serves a critical role by introducing subtle, structured perturbations into the visual space of the QR code, which are invisible to the human eye but detectable by the trained neural network.

#### 4.1.2. The Proposed Authentication Method

The generated QR code was verified using the proposed authentication method, which relies on a neural network object detection classifier. This object detection model is capable of identifying the barcode and determining the market to which it belongs. The specifics of this detection process are explained as follows:

- Object Detection

Object detection is an extension of the classification problem and is defined as a computer vision task that identifies instances of objects belonging to a specific class within images. An object detection model is trained to recognize the presence of these objects in the images.

- TensorFlow Object Detection

TensorFlow is an open-source platform developed by Google for machine learning. In object detection, TensorFlow generates a bounding box around the detected object when an image is input into the model. This bounding box includes a score that reflects the confidence level of the detection’s accuracy. In our work, we set the threshold for this score to 99%, and only if the confidence score met this threshold could the barcode authentication process proceed. For instance, in [36], a card detection classifier was developed using TensorFlow’s object detection capabilities.

#### 4.1.3. Requirements

There are three main types of system requirements: generating a secure QR code, data training, and QR code scanning and authentication. Python 3.7 was used for all these processes. For generating a secure barcode, we applied the MyQR library (version: 2.3.1) and a unique pattern (i.e., a specific digital watermark embedded within the QR code to uniquely identify the market that generated it). For data training, we set up a Conda virtual environment and utilized the TensorFlow 1.14 library, TensorFlow Object Detection 1.13 API, and the Faster_rcnn_inception_v2 model. We also used a printer, using cameras integrated in iPhone 11 and iPhone 12 smartphones (Apple Inc., Cupertino, CA, USA), and the LabelImg tool (version: 1.8.6) to prepare the dataset. For barcode scanning and authentication, we employed a Conda virtual environment, the OpenCV library, the Dynamsoft Barcode Reader SDK, and the EpocCam HD iPhone application for real-time barcode reading.

### 4.2. QR Code Dataset Preparation and Generation

The dataset used in our experiments comprises both original and forged QR code images. To simulate real-world conditions and test the system’s resilience, we included a diverse range of image variations, such as the following:

- Noisy images, by adding Gaussian noise using OpenCV during preprocessing.
- Blurred images, using random Gaussian blur filters.
- Rotated images at angles of 90°, 180°, and 270°.
- Low-resolution scans to mimic quality degradation in printed materials.

To generate realistic printed and scanned QR code samples, the required steps are described as follows:

- We used HP LaserJet Pro MFP M28w (HP Inc., Palo Alto, CA, USA) and Canon PIXMA TR4540 (Canon Inc., Tokyo, Japan) printers to print the QR codes on standard A4 paper.
- QR codes were then captured using a Samsung Galaxy S20 (Samsung Electronics Co., Ltd., Suwon, Republic of Korea) smartphone camera (12 MP) and a Logitech C920 HD webcam (Logitech International S.A., Lausanne, Switzerland) under indoor lighting conditions.
- Scanning distances and angles were varied slightly during image acquisition to mimic different usage environments.

**Figure 2:**
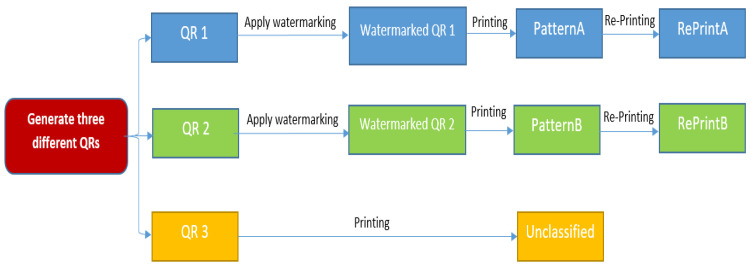
Phases of dataset preparation.

**Figure 3:**
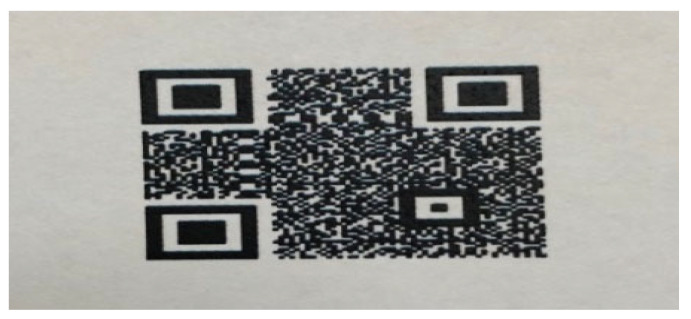
A sample of printed QRs.

The dataset preparation consisted of several phases, as illustrated in Figure 2. Specifically, we generated three QR classes using Python and the MyQR library: PatternA, PatternB, and Unclassified. The first two QRs were watermarked to represent two different markets, while the third one was left unwatermarked. These QRs were printed, as shown in Figure 3, to simulate a real-life scenario for model training. The first two QRs were then reprinted to create the PatternA_reprinted and PatternB_reprinted classes. This step mimicked a fraud case where a barcode is captured and reprinted for illegal use.

**Figure 4:**
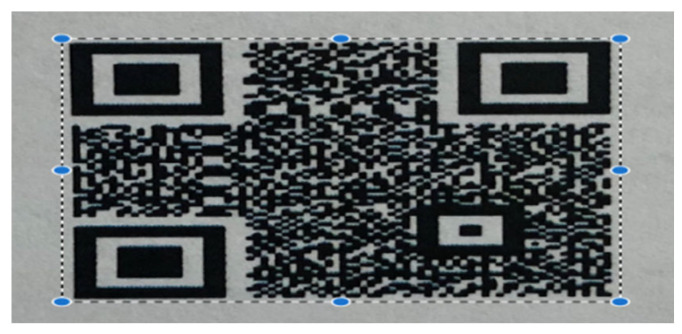
Process of Cropping the QR Codes.

The data from the reprinted QRs were prepared by manually cropping them, as shown in Figure 4. Based on this, we created five classes for training the model, as outlined in Table 2.

It is important to note that the dataset images for all five classes were varied to include all possible QR scan orientations, with angles of 0°, 90°, 180°, and 270°. The images for each class were divided equally among the different angles. The labeling process for both the printed and reprinted QRs across the five classes was carried out using the LabelImg tool.

### 4.3. Workspace Configuration

Before starting the model training process, we prepared the environment and downloaded the necessary libraries and dependencies. The process was as follows: first, we installed and activated the Conda virtual environment. Then, we installed Python 3.7, the OpenCV library, and TensorFlow 1.14. The TensorFlow API provides pre-trained models that have been trained on large datasets, and we selected a model for its execution speed and accuracy. Specifically, we chose the Faster_RCNN_Inception_V2 model to build our object detector. Afterward, we downloaded the TensorFlow Object Detection 1.13 API and installed all the required dependencies, including Pillow, lxml, cython, contextlib2, matplotlib, pandas, and pycocotools.

For the hyperparameters and model settings, the details are as follows:

- Learning rate: 0.001
- Batch size: 32
- Optimizer: Adam
- Number of convolutional layers: 5
- Activation function: ReLU
- Confidence threshold for authentication: 99%
- Epochs: 50
- Loss function: categorical cross-entropy

### 4.4. Dataset: Training and Testing

The final dataset consisted of 5000 images, with 1000 images for each class (Class 1, Class 2, Class 3, Class 4, and Class 5). We used 80% of the dataset for training and 20% for testing, as recommended in [37]. Specifically, 800 barcodes from each class were used for training, and 200 for testing, resulting in a total of 4000 training barcodes and 1000 testing barcodes. Using the neural network, the barcode classifier model was trained with approximately four times the amount of loss, which indicates how inaccurate the model’s prediction was for a single example. A perfect prediction results in a loss of zero; otherwise, the loss value is greater. Training on a CPU device took about 66 h, spanning approximately 72,260 steps, until the loss dropped below 0.05, which is the recommended threshold for the Faster_RCNN_Inception_V2 model.

### 4.5. Classifier–Reader Integration

As mentioned earlier, we used a Dynamsoft SDK reader to complete the system integration process. The system authenticates the QR using the QR classifier model. For instance, to authenticate a scanned QR with an “A” watermark pattern (i.e., PatternA), we set the minimum confidence threshold to 0.99 for PatternA. If a QR met or exceeded this confidence threshold, it was considered valid for barcode reading authentication. This authentication process is represented by the following Python code condition, which the system relies upon to ensure proper authentication, while also preventing QR reprinting.

### 4.6. Evaluation and Analysis

We evaluated the developed system by conducting two tests: QR classifier model testing and classifier–reader testing.

#### 4.6.1. QR Classifier Model Testing

**Figure 5:**
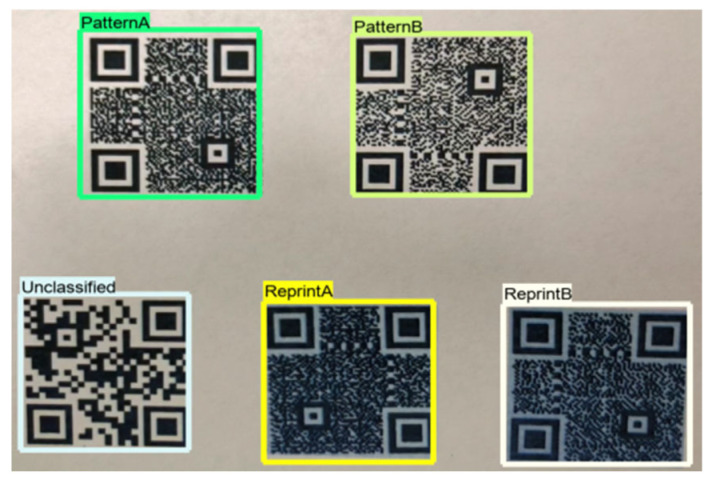
Successful five-class classification.

**Figure 6:**
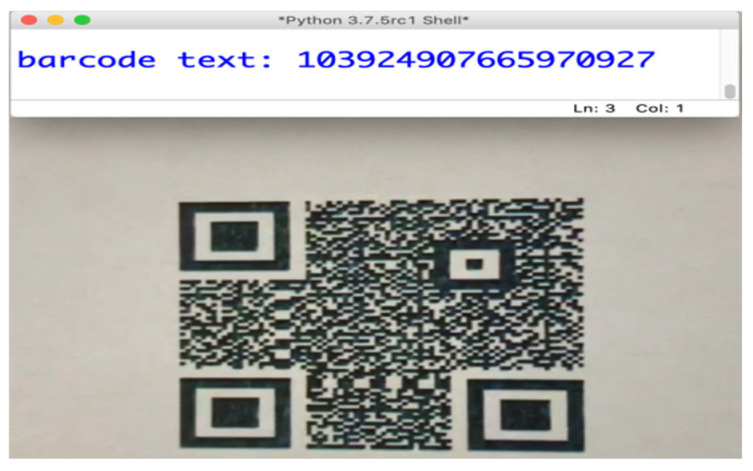
Successful QR reading.

We used a Python code in conjunction with the EpocCam HD application to ensure the QR classifier model ran in real-time. Figure 5 illustrates a successful model classification across the five classes. The height between the camera and the QR was considered an important factor influencing the QR authentication process. For example, the QR in Figure 6 was captured at a height of 3.5 inches between the iPhone camera and the QR, which was approximately 1 × 1 inch in size and had various scan orientation angles.

#### 4.6.2. Classifier–Reader Testing

**Figure 7:**
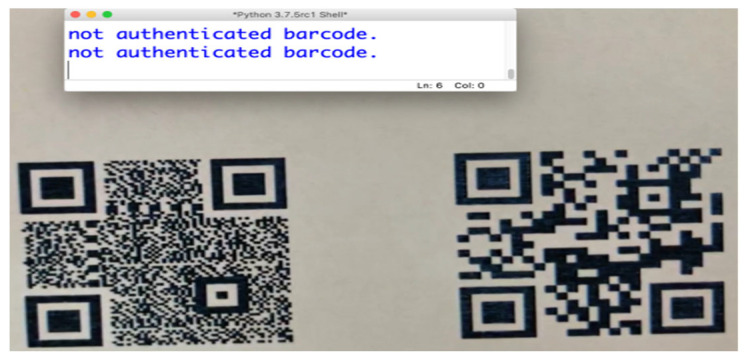
Successful QR ignoring.

We evaluated the system in this test under similar circumstances, using the same tools as in the QR classifier model test, but with an additional Python code to fulfill the requirements for this stage. Figure 6 shows the successful reading of a QR code with PatternA (as determined earlier), while Figure 7 demonstrates the successful rejection of QRs with PatternB and Unclassified. Therefore, the proposed system was able to correctly identify the authorized QR and ignore any unauthorized ones. This approach helps mitigate financial losses and reduces the need for manual checking, allowing sectors (i.e., types of shop owners or commercial categories, e.g., retail, electronics, and groceries, who utilize QR codes for their product labeling and authentication) to trust their systems to protect against the aforementioned attacks using the proposed secure QR.

### 4.7. Evaluation Metrics

To assess the model’s effectiveness, the following metrics were used:

- **Accuracy**: Overall correctness of predictions.
- **Precision**: Correctly predicted tampered codes over all predicted tampered codes.
- **Recall (Sensitivity)**: Correctly predicted tampered codes over all actual tampered codes.
- **F1-Score**: Harmonic mean of precision and recall.
- **Confusion Matrix**: For a detailed breakdown of true/false positives and negatives.

These metrics ensure a comprehensive evaluation of the classifier’s performance under realistic conditions.

### 4.8. Experimental Validity and Ethical Considerations

The methodology was designed to reflect practical scenarios where QR code misuse is likely (e.g., retail fraud). Since only synthetic data was used and no personal information was processed, no ethical approval was required.

## 5. Results

The proposed secure QR verification system was evaluated using the dataset of 5000 QR codes described earlier, consisting of both genuine and tampered samples. The model’s performance was assessed using standard classification metrics and is summarized below.

### 5.1. Classification Performance

The CNN model achieved robust classification results on the test set. Table 3 presents the key performance metrics.

These results indicate that the model is highly effective at distinguishing between authentic and tampered QR codes, with strong precision and recall values that highlight both its reliability and sensitivity.

### 5.2. Confusion Matrix

To gain more insight into the model’s predictive behavior, a confusion matrix was generated, as shown in Table 4.

The confusion matrix confirms the model’s low false-positive and false-negative rates, further validating its robustness under realistic tampering scenarios.

### 5.3. ROC Curve and AUC

**Figure 8:**
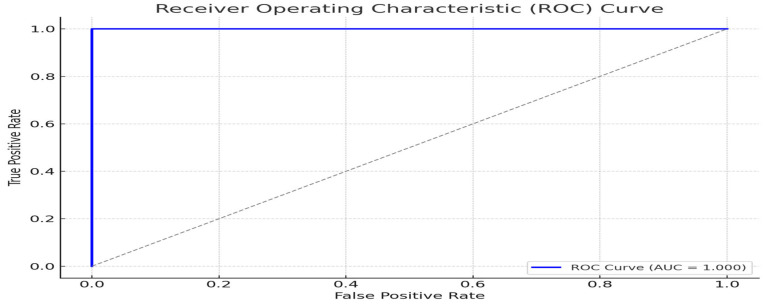
Receiver Operating Characteristic (ROC) curve.

Figure 8 shows the Receiver Operating Characteristic (ROC) curve. The Area Under the Curve (AUC) was calculated at 0.991, reflecting excellent discrimination between genuine and tampered QR codes. The curve shows a steep ascent toward the top-left corner, indicating high sensitivity and specificity.

### 5.4. Robustness to Real-World Distortions

To assess the model’s reliability in practical use cases, additional testing was performed on QR codes captured under varying lighting conditions, angles, and resolutions. Table 5 summarizes the model’s performance across these challenging environments.

The results demonstrate that the model maintains high accuracy across a range of realistic distortions, making it suitable for real-time deployment using smartphone cameras or retail scanners.

### 5.5. Processing Speed

To validate the system’s suitability for real-time applications, we evaluated its average inference time. The model processed each QR image in 24 milliseconds, enabling 40+ verifications per second on standard hardware (Intel i7 CPU, GTX 1660 GPU). This affirms the method’s practicality for integration into point-of-sale and mobile applications.

## 6. Discussion

This section analyzes the experimental findings, compares the proposed approach with the existing work, and reflects on the current limitations and potential avenues for future research in secure QR code authentication systems.

### 6.1. Interpretation of Results

The experimental evaluation revealed the high effectiveness of the proposed system in detecting tampered QR codes, with a classification accuracy of 97.2% and an AUC of 0.991. These results confirm the model’s robustness in real-world conditions, including low lighting, skewed angles, and resolution loss.

Interestingly, the model trained exclusively on real-life QR images outperformed the model trained on a mix of synthetic and captured codes. This highlights the importance of training with representative real-world data for deployment-oriented systems. Moreover, augmenting the dataset with multiple orientations (0°, 90°, 180°, and 270°) significantly improved the detection accuracy, confirming the relevance of orientation-aware training.

We also observed that using watermarked patterns with visually distinct designs improved classification precision but slightly reduced authentication security. This highlights a practical trade-off: more visually similar patterns enhance authentication robustness but make class separation more difficult for the model. Future efforts will explore methods to dynamically balance this trade-off.

Finally, incorporating reprinted QR codes—representing fraudulent duplicates—proved beneficial in training the model to recognize unauthorized reissues. This demonstrates the model’s capability not only to authenticate legitimate codes but also to detect suspicious duplications or tampering.

Notably, the inclusion of reprinted classes, which acted as fraudulent QRs, enhanced the security level of the proposed system. As a result, when the model classifies a QR as belonging to a specific market, it ensures that the QR was originally printed by that market and has not been reprinted or tampered with by any external party.

### 6.2. Comparison with the Existing Literature

Most prior studies on QR security have focused on detecting malicious links using standard URL classification datasets, e.g., [38,39,40,41,42,43]. Unlike those approaches, our work introduces a watermarking-based tamper-proof mechanism embedded directly in the QR visual pattern and leverages a neural network to authenticate it without needing external fingerprint databases or cloud-based lookup.

Compared to systems using blockchain [19,22] or mathematical secure algorithms [29], our framework offers a lightweight, localized solution that eliminates the dependency on third-party infrastructure. Furthermore, unlike [36], our model is designed to function under constrained computing environments with near real-time processing speeds. Table 6 presents a comparative analysis of our model against existing studies. 

### 6.3. Limitations and Challenges

Although we successfully integrated the QR classifier model with a commercial Dynamsoft SDK reader, which is capable of decoding information, we encountered issues with the reading process not being as fast as anticipated. Challenges also arose when attempting to read the QRs intermittently. Additionally, several QR reader libraries struggled to decode the proposed secure QRs due to the detailed watermark patterns embedded within them. Nonetheless, the primary aim of our work was to enhance the security of QRs and make them more resistant to fraud.

Moreover, there was a challenge in adopting the chosen patterns due to the inverse relationship between similar and dissimilar appearances, which impacted the balance between security and classification precision. Additionally, it proved difficult to find a reader capable of decoding the information on the proposed secure barcode, as several QR reader libraries were unable to process it. To mitigate this, we used the commercial Dynamsoft SDK reader, although its performance was not as fast as anticipated, and issues arose when reading QRs intermittently.

Furthermore, it would be valuable to investigate the impact of QR code orientation on detection accuracy and classification performance, as well as to develop orientation-invariant features or preprocessing techniques to enhance system robustness. In addition, it would be beneficial to assess the system’s ability to handle real-world scenarios in which QR codes are scanned from various angles.

We believe these future efforts will provide valuable insights and ensure the system’s applicability in diverse environments.

These limitations and challenges are summarized as follows:

- Reading performance: Integration with a commercial reader (Dynamsoft SDK) was successful, but read speeds were slower than expected, especially under variable scanning angles or lighting.
- Reader compatibility: Several standard QR libraries failed to decode our secure QR codes due to the watermarking patterns embedded in the structure, requiring reliance on commercial SDKs.
- Pattern design trade-off: Achieving optimal security often required visually similar patterns, which occasionally impacted model precision. Designing patterns that balance robustness and class separability remains a challenge.
- Orientation variance: Though our multi-angle training improved the accuracy, further enhancements are needed for dynamic orientation-invariant QR decoding in real-time scenarios.

### 6.4. Future Research Directions

Based on the findings and challenges, we identify several directions for future work, which are as follows:

- Improve the decoding speed of secure QR codes and reduce latency in reader integration.
- Design or adapt an open-source QR reader tailored to handle watermark-embedded secure QR codes.
- Expand the dataset to include more diverse QR designs and simulate complex tampering attacks for deeper resilience testing.
- Compare the proposed system with additional state-of-the-art models using large-scale public QR datasets.
- Investigate more advanced neural network architectures, such as transformers or hybrid CNN-RNN models, for more scalable and orientation-invariant classification.

## 7. Conclusions

This study proposed a secure and efficient QR code authentication framework that combines visual watermarking with convolutional neural networks (CNNs) to verify the authenticity of printed QR codes and detect tampering—without relying on cloud-based databases or stored QR fingerprints. The system achieved a high classification accuracy of 97.2% and demonstrated strong performance under various real-world scanning conditions. By training on real-life data and incorporating orientation-aware images, the model proved robust against common distortions encountered during retail or mobile scanning. The use of embedded watermark patterns enabled localized, market-specific verification, adding a critical layer of protection against counterfeit duplication. The integration of AI in the form of a CNN classifier represents a significant advancement in QR code authentication. It enables the system to learn complex visual patterns and make accurate authentication decisions locally, offering a scalable and intelligent solution that addresses modern security challenges. Overall, this work advances the development of intelligent, tamper-resistant QR authentication systems and lays the foundation for secure deployment in privacy-sensitive, high-security smart environments.

Unlike traditional approaches, the proposed method supports fast, offline authentication suitable for resource-constrained environments, such as point-of-sale systems and smart retail settings. The integration of neural networks ensures real-time processing, enhancing both practicality and scalability. Despite these promising results, some challenges remain—particularly in optimizing reader compatibility, improving decoding speed, and managing the trade-off between watermark similarity and classification accuracy. Future research will address these issues by expanding the dataset, refining the watermark design, and exploring more advanced machine learning models.

## Figures and Tables

**Table 1:** Original barcode samples vs. regenerated ones.

No	Original Barcode Sample	Regenerated Barcode Sample	Used Tool
1	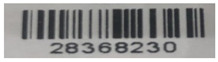	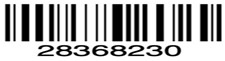	iBarcoder
2	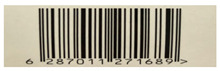	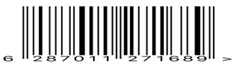	Easy Barcode Creator
3	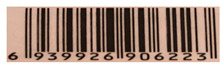	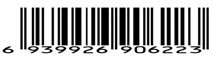	Easy Barcode Creator
4	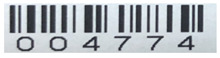	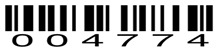	TEC-IT
5	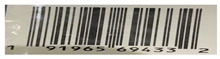	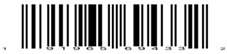	TEC-IT

**Table 2:** Classes and their samples.

	Class Name				
	PatternA	ReprintA	PatternB	ReprintB	Unclassified
Class sample	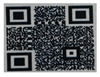	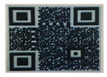	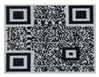	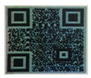	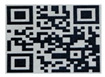

**Table 3:** Performance of QR code classification model.

Metric	Value (%)
Accuracy	97.2
Precision	96.8
Recall	97.6
F1-Score	97.2

**Table 4:** Confusion matrix for test set predictions.

	Predicted Genuine	Predicted Tampered
Actual Genuine	732	18
Actual Tampered	14	736

**Table 5:** Model accuracy under varying image conditions.

Condition	Accuracy (%)
Normal lighting	97.6
Low lighting	95.2
Skewed angle (≤15°)	96.1
Reduced resolution	94.7

**Table 6:** Comparative analysis with related previous studies.

Study	Year	Approach
[17]	2024	Blockchain
[19]	2023	Deep Neural Network (DNN)
[22]	2023	Blockchain
[29]	2024	Mathematically Secure Computational Algorithms
[36]	2024	Adaptive Morphological QR Extraction (AMCQE)
Our study	2025	Watermarking + Neural Network

## Data Availability

The data that support the findings of this study are available from the corresponding author, Suliman A. Alsuhibany, upon reasonable request.
